# Alleviating occupational stress in Chinese junior high school teachers: the role of mindfulness-based interventions

**DOI:** 10.3389/fpsyg.2025.1479507

**Published:** 2025-01-29

**Authors:** Huimian Bian, He Jiang

**Affiliations:** ^1^Wudong Hospital, Wuhan, Hubei, China; ^2^Faculty of Social Sciences and Liberal Arts, UCSI University, Kuala Lumpur, Malaysia; ^3^School of Music and Dance, Guangzhou University, Guangzhou, China

**Keywords:** educational reform, junior high school teachers, mindfulness, occupational stress, mental health, quality education

## Abstract

**Introduction:**

Occupational stress is a significant issue among junior high school teachers in China, contributing to negative outcomes such as reduced mental health, impaired coping abilities, and decreased job satisfaction.

**Methods:**

This quasi-experimental study investigates the impact of mindfulness-based interventions on occupational stress and mental health among junior high school teachers in China. A total of 118 teachers participated in the study, with a randomly assigned experimental group undergoing an 4-week mindfulness training program, while the control group received no intervention. Standardized measures of occupational stress, mental health, coping self-efficacy, and mindfulness were used to assess the outcomes before and after the intervention.

**Findings:**

The findings revealed that teachers who participated in the mindfulness program experienced significant reductions in occupational stress and improvements in mental health and coping self-efficacy compared to the control group. Additionally mindfulness levels increased significantly among participants who underwent the training.

**Discussion:**

The results suggest that mindfulness-based interventions can effectively alleviate occupational stress and enhance psychological wellbeing among junior high school teachers in China, highlighting the importance of implementing such programs to support educators in managing stress and maintaining mental health.

## 1 Introduction

Teachers' mental health is a crucial indicator of their teaching quality, significantly influencing both their career development and students' psychological wellbeing (Benevene et al., 2020). Reports indicate that 60%–70% of teachers worldwide experience significant psychological stress due to long-term heavy teaching tasks, strained interpersonal relationships, poor working conditions, and low compensation (Wettstein et al., 2023; Rashid, 2022). This stress can lead to severe physical and mental health issues, adversely affecting teachers' personal and professional lives (Wettstein et al., 2023).

In China, multiple studies have highlighted the prevalence of psychological problems among primary and junior high school teachers (Wang et al., 2015). Therefore, it is of utmost importance to address the mental health of Chinese teachers. Junior high school teachers, in particular, face additional professional pressures due to an increasingly diverse student population and declining parental involvement. These high stress levels lead to decreased productivity, health problems, and increased turnover, negatively impacting educational stability and quality (Clunies-Ross et al., 2008; Montgomery and Rupp, 2005).

Most studies examining the occupational stress and mental health of junior high school teachers focus on organizational factors such as work environment, job stress, and social support (Chennoufi et al., 2012; Gluschkoff et al., 2016). However, there is a relative paucity of research on the role of individual characteristics (Rey et al., 2016; Kim et al., 2019). Schwarzer and Hallum (2008) suggest that low self-efficacy may increase job stress and susceptibility to burnout. Conversely, mindfulness has been shown to be closely related to reducing occupational stress and improving physical and mental health among teachers (Sun et al., 2019).

This study aims to fill the gap by focusing on the individual characteristics of mindfulness and its impact on occupational stress among junior high school teachers in China. This study designed an effective Mindfulness Programme for junior high school teachers' occupational stress and mental health. The primary objective was to evaluate the impact of a mindfulness program on occupational stress, mental health, coping self-efficacy, and mindfulness levels among junior high school teachers. There are two research questions in this study:

1. What are the changes in coping self-efficacy, mental health, occupational stress, and mindfulness levels among junior high school teachers before and after participating in the Mindfulness Programme?
2. What are the differences in the levels of coping self-efficacy, mental health, occupational stress, and mindfulness between the group of junior high school teachers who received the Mindfulness Programme and those who did not?

This research offers a novel approach to improving teachers' mental health by addressing the individual characteristics of junior high school teachers and providing a targeted intervention. The findings are expected to provide valuable insights for educational policymakers and practitioners seeking to enhance teacher wellbeing and, consequently, the quality of education.

## 2 Literature review

### 2.1 Mindfulness and mental health

Mindfulness is defined as paying full attention to the present moment experience in a deliberate and non-judgmental way (Bishop et al., 2004). It comprises two main aspects: self-regulation of attention and orientation to experience. The former involves an individual's ability to stay focused, reduce distracting thoughts, and increase awareness of inner experiences, thereby enhancing emotional regulation capabilities. The latter refers to adopting a specific attitude toward current experiences, which aids in managing emotions and coping with stress (Bergomi et al., 2013).

Mindfulness training has been shown to positively impact teachers' mental health by reducing stress, enhancing wellbeing, and improving emotional regulation. This is supported by various studies conducted in different educational and cultural contexts (Janssen et al., 2023; Morales-Rodríguez and Morales-Rodríguez, 2024). Mindfulness helps teachers consciously select and identify their thoughts, emotions, and feelings in the work environment, reducing habitual emotional and behavioral responses. It also allows teachers to mentally detach from work during leisure time, thus perceiving, experiencing, and expressing their emotions more fully, reducing stress perception and the accumulation of negative emotions (Nevill and Havercamp, 2019).

Mindfulness training has been shown to significantly reduce job burnout and work stress, thereby improving depression, anxiety, and overall mental health among teachers (Dave et al., 2020; Tsang et al., 2021). Therefore, it is hypothesized that mindfulness is positively related to the mental health of junior high school teachers.

### 2.2 Occupational stress and treatment for teachers

Occupational stress among teachers is a significant issue that affects their wellbeing and performance. It arises from various factors, including high job demands, emotional challenges, and inadequate support, leading to burnout and reduced occupational wellbeing. Effective treatments and interventions are crucial to mitigate these stressors. Occupational stress may play a critical mediating role in the relationship between mindfulness and mental health. Janssen et al. (2023) found that Mindfulness-Based Stress Reduction combined with organizational health interventions can protect individuals against emotional and mental health problems, potentially mitigating the effects of these issues. Similarly, Candeias et al. (2024) suggest developing effective interventions and strategies to promote optimal health, wellbeing, and resilience. Starr (2023) found that mindfulness interventions aimed at reducing work stress improved emotional regulation in early childhood teachers, which in turn positively impacted their mental health.

Occupational stress among teachers is a multifaceted issue stemming from various factors. High job demands coupled with emotional challenges pose significant contributors to stress and burnout among educators (Fitchett et al., 2019; Nwoko et al., 2023). Additionally, the workplace environment plays a crucial role; a poor school climate and inadequate resources exacerbate stress levels (Jõgi et al., 2022). Furthermore, the COVID-19 pandemic has intensified this stress due to heightened job demands and associated health risks, further illustrating the complexity and urgency of addressing occupational stress in the teaching profession (Tesfaye et al., 2023).

Treatments and interventions for reducing occupational stress among teachers encompass a range of approaches. Cognitive Behavioral Therapy, particularly when combined with relaxation techniques, has demonstrated significant efficacy in alleviating stress (Denuwara et al., 2021). Online Cognitive Behavioral Interventions have also proven effective, particularly among ESL teachers, and are well-regarded (Eze et al., 2023). Standalone relaxation interventions and meditation offer additional stress-reducing benefits, with meditation being another notable intervention (Paudel et al., 2022). Overall, a combination of these methods often yields the most favorable outcomes in managing stress among teachers.

Enhancing occupational wellbeing among teachers involves nurturing socioemotional competence, which aids in better stress management and fosters a positive classroom environment (Nwoko et al., 2023). Additionally, organizational support from school leadership and stakeholder collaboration play a pivotal role in boosting teachers' resilience and job satisfaction (Nwoko et al., 2023). Furthermore, teachers who possess high self-efficacy and have access to sufficient resources typically experience lower stress levels (Jõgi et al., 2022), underscoring the importance of these factors in promoting wellbeing in teaching.

### 2.3 Teachers' coping self-efficacy and interventions

Teachers' coping self-efficacy refers to their belief in their ability to manage stress and challenges effectively in their professional environment. Interventions aimed at enhancing this self-efficacy can significantly impact teachers' mental health, job satisfaction, and overall wellbeing. Coping self-efficacy is a crucial personal resource for teachers, helping them manage stress and maintain mental health. It is linked to better psychological resilience and positive emotions, which are essential for a successful teaching career (von Muenchhausen et al., 2021; Won and Chang, 2019). Personality traits such as conscientiousness, openness, and self-efficacy are significant predictors of proactive coping strategies. These strategies include reflective, strategic planning, and preventive coping, which can be learned and enhanced through training programs (Samfira and Paloş, 2021; Mikus and Teoh, 2021).

Interventions designed to enhance self-efficacy have demonstrated a substantial positive impact on promoting teacher self-efficacy, particularly those incorporating mastery experiences, which are highly effective for pre-service teachers. Various types of interventions exist, including psychological group programs that improve mental health and self-efficacy, leading to heightened life satisfaction and improved coping strategies (von Muenchhausen et al., 2021). Online stress interventions are also effective in reducing burnout and enhancing teacher efficacy by imparting coping strategies and fostering social-emotional competencies (Ansley et al., 2021). Furthermore, mindfulness and compassion training programs, such as the “Call to Care,” emphasize mindfulness and compassion, thereby improving teachers' sense of efficacy and alleviating stress (Tarrasch et al., 2020). Additionally, positive psychology interventions are utilized to bolster resilience, emotion regulation, and coping abilities, especially among pre-service teachers (Hoferichter and Jentsch, 2024).

Existing literature highlights the significant benefits of mindfulness-based interventions, occupational stress management strategies, and the enhancement of teachers' coping self-efficacy in improving mental health, increasing job satisfaction, and reducing burnout. However, these studies have predominantly focused on specific cultural or educational contexts, with limited attention to the unique challenges faced by junior high school teachers in China. Furthermore, while some research has examined the relationships between mindfulness, occupational stress, and mental health, there remains a lack of in-depth analysis of the interactive mechanisms among these variables. Notably, the influence of contextual factors such as school environments, leadership support, and cultural dimensions has been underexplored. To address these gaps, the present study seeks to investigate how mindfulness-based interventions alleviate occupational stress and enhance mental health among Chinese junior high school teachers, with particular consideration of these contextual influences.

### 2.4 Theoretical framework

The proposed framework integrates theories, empirical findings, and methodological insights from mindfulness research to investigate the impact of mindfulness-based interventions (MBIs) on occupational stress and mental health among Chinese junior high school teachers. Grounded in Shapiro et al.'s (2006) IAA model of mindfulness and incorporating Kabat-Zinn's (1994) foundational perspectives, the framework systematically addresses key constructs, their interrelations, and the contextual dynamics influencing outcomes.

The present study is grounded in key constructs and theoretical foundations, focusing on mindfulness as the independent variable. Mindfulness is defined using Shapiro et al.'s (2006) three-axis model (IAA model), encompassing intention (I), which refers to the motivation behind mindfulness training aimed at cultivating awareness and compassion, attention (A), which involves focused awareness on the present moment to reduce automatic stress-inducing reactions, and attitude (A) characterized by a non-judgmental, receptive mindset that fosters acceptance and psychological resilience. Occupational stress and mental health serve as dependent variables. Occupational stress is measured by the intensity and frequency of stressors derived from Zhu et al. (2002) model of teacher-specific stress, including workload, administrative demands, and interpersonal conflicts. Mental health is operationalized through multidimensional factors of psychological wellbeing and distress, such as those assessed by the SCL-90 dimensions. Coping self-efficacy, conceptualized based on Bandura et al.'s (2001) work as the ability to effectively manage stress and foster positive coping strategies, functions as a mediating variable, enhancing the relationship between mindfulness and reductions in occupational stress. Additionally, demographic and contextual factors, including gender, teaching experience, and professional roles, are examined as potential moderating variables influencing the relationship between mindfulness practices and their outcomes.

**Figure 1:**
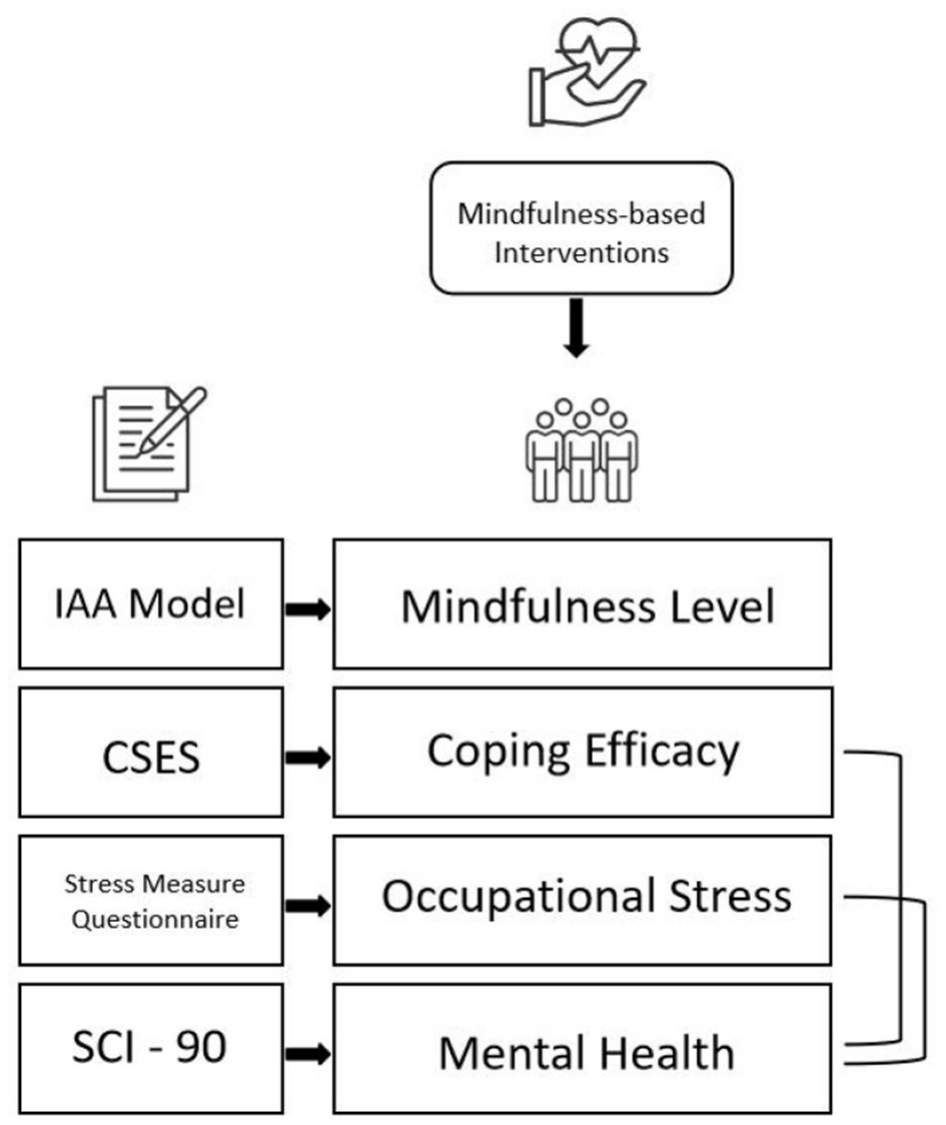
Theoretical framework for mindfulness-based interventions.

The framework structure of this study elucidates pathways of influence, contextual considerations, intervention design and implementation, and measurement and evaluation (Figure 1). Specifically, it outlines a direct pathway where mindfulness training directly reduces occupational stress through heightened self-awareness and emotional regulation and an indirect pathway where mindfulness fosters improved coping self-efficacy, which in turn mediates its effects on stress and mental health. Recognizing the unique occupational environment of Chinese junior high school teachers, the framework takes into account educational reforms, high workloads, and cultural expectations of teacher roles. Guided by the IAA model, the mindfulness program is tailored to address teacher-specific stressors across four domains: self, students, colleagues, and work, and utilizes a hybrid delivery model (offline and online) to ensure comprehensive engagement and reinforce mindfulness in real-world settings. Key variables are measured using validated instruments, and statistical analysis involves pre- and post-intervention comparisons to evaluate the efficacy of the intervention.

This framework offers a comprehensive approach to studying MBIs in the context of occupational stress, advancing both theoretical understanding and practical applications in education.

## 3 Methods

### 3.1 Research procedure

The study was conducted in several stages. Initially, participants were recruited from six junior high schools in Henan Province, China. The recruitment was facilitated through school leaders who disseminated an online survey link to potential participants. The survey assessed the teachers' baseline levels of mindfulness, occupational stress, coping self-efficacy, and mental health. Mindfulness-based cognitive therapy (MBCT) and mindfulness-based stress reduction (MBSR) techniques were employed, along with mindfulness meditation practices targeting occupational stress.

After the initial assessment, participants were randomly assigned to either the mindfulness intervention group or the control group. The intervention group participated in a structured mindfulness program over the course of 1 month, which included both offline and online components. The control group did not receive any mindfulness training during this period.

Post-intervention assessments were conducted to evaluate any changes in the levels of mindfulness, occupational stress, coping self-efficacy, and mental health. These assessments were identical to the baseline survey to ensure consistency and comparability of data.

### 3.2 Participants

This study recruited 120 junior high school teachers from various schools in China, selected randomly based on questionnaire responses. The primary inclusion criterion was the absence of prior comprehensive mindfulness training among the participants. The experimental group initially comprised 60 participants, with 2 participants dropping out (dropout rate: 3.33%), resulting in 58 individuals completing the intervention. In contrast, no participants from the comparison group (*n* = 60) dropped out, and all remained engaged throughout the study. Overall, 118 participants successfully completed all required tasks for both the offline and online components of the course.

The participant demographics were as follows: 24 males (20.3%) and 94 females (79.7%), with 56 participants aged 21–30 (47.5%), 53 participants aged 31–40 (44.9%), and 9 participants aged 41–50 (7.6%). The teaching experience of participants varied, with 17 teachers having 1–5 years of experience (14.4%), 53 teachers having 6–10 years of experience (44.9%), 47 teachers having 11–15 years of experience (39.8%), and one teacher with 16–20 years of experience (0.8%).

In terms of educational attainment, 9 participants had a junior college degree or below (7.6%), 90 had a bachelor's degree (76.3%), and 19 had a master's degree (16.1%). Regarding their professional roles, 33 participants were classroom teachers (28%), and 85 were non-classroom teachers (72%).

Comparative analysis revealed no significant differences in age, gender, educational level, teaching experience, or professional role between the control group and the treatment group, ensuring the group's comparability.

### 3.3 Intervention design

#### 3.3.1 Course rationale

The mindfulness course was designed with both offline and online components to enhance participants' mindfulness practices effectively. Its theoretical foundation is Shapiro et al.'s (2006) three-axis model of mindfulness, which identifies intention, attention, and attitude (IAA) as the core elements essential for mindfulness training. These components are indispensable and intricately connected, providing a comprehensive framework for understanding how mindfulness operates.

According to Kabat-Zinn (1994), mindfulness can be understood as the awareness that emerges through critical attention, encompassing the three elements of intention, attention, and attitude. These elements do not exist in isolation but are interwoven and occur simultaneously, forming the basis for various changes observed during mindfulness practice. Mindfulness is characterized by a state of presence in the current moment, facilitated by the harmonious interaction of these three axes.

##### 3.3.1.1 Intention (I)

This element defines the purpose and motivation behind mindfulness training. It determines the direction and intensity of change and provides criteria for evaluating its effectiveness. Mindfulness practice intends to cultivate kindness and compassion, transforming harmful and unhealthy tendencies toward others. By clarifying why mindfulness training is undertaken, intention helps guide the practitioner's focus and commitment.

##### 3.3.1.2 Attention (A)

Attention is the central component of mindfulness practice. It involves a clear and focused awareness of personal physical and mental changes while maintaining a high level of attentiveness to the external environment. Mindfulness training requires observing one's behavior and internal and external experiences in each moment, allowing individuals to develop greater self-awareness and presence.

##### 3.3.1.3 Attitude (A)

This aspect refers to a non-judgmental, receptive, friendly, and open approach to both internal and external experiences. Attitude aligns closely with the spirit of meditation, emphasizing peace, tolerance, and acceptance. Practitioners are encouraged to abandon unhealthy mentalities such as extremes, concerns, and rejection and, instead, maintain a purely receptive and accepting mindset.

Building upon the IAA model and considering the specific characteristics of occupational stress faced by Chinese junior high school teachers, the mindfulness course was structured into four distinct units: self, students, colleagues, and work. Each unit addresses a different aspect of the participants' professional lives, aiming to equip them with the skills and perspectives necessary to manage stress and foster a more mindful approach to their roles.

#### 3.3.2 Course duration and mode

The mindfulness course was conducted over 4 weeks and comprised both offline and online components designed to provide a comprehensive training experience. The course was structured to balance in-person instruction with daily individual practice, enabling participants to integrate mindfulness into their daily lives effectively.

##### 3.3.2.1 Offline course

Duration and Frequency: The offline component consisted of four face-to-face sessions held once a week over the course of a month. Each session lasted between 110 and 120 min.

Location: Sessions took place in a spacious school classroom equipped with movable tables and chairs to facilitate group activities and discussions.

##### 3.3.2.2 Online practice

Daily Mindfulness Practice: Participants engaged in daily mindfulness practice throughout the month. The daily exercises were based on the third and fourth activities introduced during the weekly offline sessions and required 15–25 min of practice per day.

Practice Environment: The online practice was designed to be conducted in a quiet, independent space free from disturbances. Participants needed access to audio equipment and a mobile device with the Qingxi app to follow guided meditation exercises.

This hybrid course design allowed participants to benefit from direct instruction and group interaction during the offline sessions while reinforcing their learning and mindfulness skills through regular, structured practice in their environments.

#### 3.3.3 Course contents and structure

Appendix 1 shows the actions taken in the Mindfulness course based on MBSR and MBCT (Kabat-Zinn, 2003; Segal et al., 2002). These Actions can be coordinated with each other or performed independently. Appendix 2 demonstrates the content of mindfulness teaching.

### 3.4 Measurements

The validated instruments were employed to measure the key variables in this study:

Mindful Attention Awareness Scale (MAAS): This scale measures mindfulness and consists of 15 items rated on a six-point Likert scale. Higher scores indicate higher levels of mindfulness. The Chinese version of MAAS demonstrated good psychometric properties, with a Cronbach's α coefficient of 0.84. Typical items include “I find it difficult to stay focused on what is happening in the present,” “I rush through activities without being really attentive to them,” and “I snack without being aware that I am eating,” reflecting habitual tendencies to operate on “automatic pilot” rather than with conscious awareness.

Question about Occupational Stress of Primary and Junior High School Teachers Questionnaire: Developed by Zhu et al. (2002), this questionnaire includes 46 items measuring total stressors, total occupational stress intensity, and six sub-dimensions. The internal consistency coefficient is 0.94. Example items include “There are too many students in the class,” “Low pay and financial embarrassment,” and “Parents of students have high expectations of teachers,” with responses rated on a scale from 0 (not at all) to 4 (extremely).

Coping Self-Efficacy Scale (CSES): This scale assesses coping self-efficacy through 26 items measuring problem-focused coping, receiving social support, and stopping unpleasant emotions and thoughts. The internal consistency was excellent (α = 0.91). Example items include “Think about one part of the problem at a time,” “Do something positive for yourself when you are feeling discouraged,” and “Resist the impulse to act hastily when under pressure,” rated on a scale from 0 (cannot do it) to 10 (can do it).

Symptom Checklist 90 (SCL-90): This scale measures mental health across nine symptom factors using a 5-point Likert scale. Higher scores indicate greater distress or dysfunction. The reliability and validity of the Chinese version are well-established, with homogeneity reliability above 0.69 and test-retest correlation above 0.73. Typical items include “Headaches,” “Nervousness or shakiness inside,” “Trouble falling asleep,” “Feeling blue,” and “Pains in heart or chest,” covering a wide range of psychological, emotional, and somatic issues.

### 3.5 Data collection and analysis

The data analysis for this study was conducted using IBM SPSS Statistics version 26.0. The primary objective was to evaluate the impact of a mindfulness program on occupational stress, mental health, coping self-efficacy, and mindfulness levels among junior high school teachers. The analysis included both within-group and between-group comparisons to assess the efficacy of the mindfulness intervention.

Descriptive statistics were computed for all study variables to summarize the demographic characteristics of the sample and to examine baseline equivalence across the experimental and control groups. Means and standard deviations were calculated for each outcome variable, including occupational stress, mental health, coping self-efficacy, and mindfulness levels.

Independent samples *t*-tests were employed to assess the differences between the experimental and control groups. The analysis was conducted to determine:

Baseline Equivalence: Independent samples *t*-tests were performed on pre-test scores for both the experimental and control groups to ensure that there were no significant differences in occupational stress, mental health, coping self-efficacy, and mindfulness levels before the intervention. The results indicated non-significant differences, confirming baseline equivalence.

Post-Intervention Comparisons: Post-intervention *t*-tests were conducted to compare the experimental and control groups on all outcome measures. Significant differences between groups were interpreted as evidence of the intervention's effectiveness.

Paired samples *t*-tests were conducted within the experimental group to evaluate the changes from pre- to post-test in occupational stress, mental health, coping self-efficacy, and mindfulness levels. This analysis assessed the magnitude and significance of the changes attributable to the mindfulness intervention.

For each *t*-test, 95% confidence intervals were calculated to estimate the precision of the mean differences. Additionally, effect sizes were computed to quantify the magnitude of the intervention effects. Cohen's *d* was calculated for paired and independent samples *t*-tests, with values interpreted as small (*d* = 0.2), medium (*d* = 0.5), and large (*d* = 0.8) effects, respectively.

Statistical significance was set at *p* < 0.05 for all analyses. Results with *p*-values < 0.001 were considered highly significant, indicating a strong effect of the mindfulness intervention.

By employing these statistical analyses, the study aimed to provide a comprehensive assessment of the mindfulness program's impact on reducing occupational stress and enhancing mental health, coping self-efficacy, and mindfulness levels among junior high school teachers.

## 4 Results

### 4.1 Results for research question 1

The study aimed to evaluate the impact of the Mindfulness Programme on occupational stress, mental health, coping self-efficacy, and mindfulness levels among junior high school teachers. Table 1 presents the results of the independent samples *t*-test for the experimental group.

**Table 1:** Results of independent samples *t*-test for the experimental group.

		**Mean**	**SD**	**SEM**	**95% Confidence interval of the difference**		***t***	**df**	**Sig**.
					**Lower**	**Upper**			
Pair 1	Occupational stress pretest—Occupational stress post-test	19.913	3.672	0.482	18.948	20.879	41.301	57	0.000
Pair 2	Mental health pretest—Mental health post-test	18.896	14.891	1.955	14.981	22.811	9.664	57	0.000
Pair 3	Coping self-efficacy pretest—Coping self-efficacy post-test	−11.137	3.491	0.458	−12.056	−10.219	−24.294	57	0.000
Pair 4	Mindfulness level pretest—Mindfulness level post-test	−7.258	6.100	0.801	−8.862	−5.654	−9.062	57	0.000

SD, Standard Deviation; SEM, Standard Error of the Mean; t, t-test value; df, Degrees of Freedom; Sig., Significance.

The results for occupational stress revealed a significant decrease in scores from the pretest to the post-test. The mean difference was 19.913 (SD = 3.672), with a standard error of the mean (SEM) of 0.482. The 95% confidence interval for the difference ranged from 18.948 to 20.879. The *t*-test yielded a *t*-value of 41.301 with 57 degrees of freedom (df), and the results were statistically significant (*p* < 0.001). This indicates that the Mindfulness Programme significantly reduced occupational stress among the participants.

Similarly, there was a significant improvement in mental health scores post-intervention. The mean difference was 18.896 (SD = 14.891), with an SEM of 1.955. The 95% confidence interval for the difference was between 14.981 and 22.811. The *t*-value was 9.664 with 57 degrees of freedom, and the results were statistically significant (*p* < 0.001). This suggests that the Mindfulness Programme effectively enhanced mental health outcomes.

In terms of coping self-efficacy, the results indicated a significant increase in scores from the pretest to the post-test, as reflected by a mean difference of −11.137 (SD = 3.491) and an SEM of 0.458. The 95% confidence interval for the difference ranged from −12.056 to −10.219. The *t*-value was −24.294 with 57 degrees of freedom, and the results were statistically significant (*p* < 0.001). This finding demonstrates that the Mindfulness Programme significantly boosted coping self-efficacy among the teachers.

Finally, the mindfulness levels showed a significant increase following the intervention. The mean difference was −7.258 (SD = 6.100), with an SEM of 0.801. The 95% confidence interval for the difference extended from −8.862 to −5.654. The *t*-value was −9.062 with 57 degrees of freedom, and the results were statistically significant (*p* < 0.001). These results highlight the efficacy of the Mindfulness Programme in enhancing mindfulness levels.

Overall, the Mindfulness Programme led to significant improvements across all measured outcomes, indicating its effectiveness in reducing occupational stress, improving mental health, and enhancing both coping self-efficacy and mindfulness among junior high school teachers.

### 4.2 Results for research question 2

Table 2 assesses the impact of a mindfulness intervention on occupational stress, mental health, coping self-efficacy, and mindfulness levels among junior high school teachers. Independent samples *t*-tests compare pre- and post-test scores between the experimental and control groups.

**Table 2:** Results of independent samples *t*-test for pre- and post-tests in experimental and control groups.

	**Levine's test of variance equivalence**		**Mean equivalence** ***t*****-test (equal variances assumed)**						
	**F**	**Sig**.	***t***	**df**	**Sig**.	**Mean**	**SEM**	**95% Confidence interval of the difference**	
								**Lower**	**Upper**
Occupational stress pretest	0.034	0.854	−1.149	116	0.253	−5.151	4.483	−14.031	3.729
Mental health pretest	0.004	0.947	−1.701	116	0.092	−8.084	4.753	−17.498	1.331
Coping self-efficacy pretest	0.523	0.471	1.142	116	0.256	2.672	2.340	−1.962	7.307
Mindfulness level pretest	1.523	0.220	1.144	116	0.255	1.141	0.997	−0.834	3.116
Occupational stress post-test	0.175	0.677	−5.496	116	0.000	−24.215	4.406	−32.941	−15.489
Mental health post-test	0.012	0.913	−6.006	116	0.000	−25.714	4.281	−34.194	−17.234
Coping self-efficacy post-test	0.444	0.507	5.798	116	0.000	12.660	2.183	8.336	16.985
Mindfulness level post-test	0.248	0.619	6.609	116	0.000	7.466	1.130	5.229	9.703

F, F-statistic; SD, Standard Deviation; SEM, Standard Error of the Mean; t, t-test value; df, Degrees of Freedom; Sig., Significant.

Prior to the intervention, the analysis showed no significant differences between the experimental and control groups across the measured variables, indicating that the groups were comparable at baseline. Specifically, the pretest results for occupational stress revealed a non-significant *t*-value, *t*_(116)_ = −1.149, *p* = 0.253, suggesting no initial difference in stress levels between the two groups. Similarly, mental health scores did not differ significantly, *t*_(116)_ = −1.701, *p* = 0.092, with the mean difference failing to reach statistical significance, reinforcing the equivalence of the groups before the mindfulness training. Coping self-efficacy also did not differ significantly at pretest, *t*_(116)_ = 1.142, *p* = 0.256, and the mindfulness levels were similarly aligned, *t*_(116)_ = 1.144, *p* = 0.255. These findings suggest that any post-intervention differences can be more confidently attributed to the intervention rather than pre-existing disparities between the groups.

Post-intervention analysis revealed significant differences in all measured variables, indicating the effectiveness of the mindfulness program. Occupational stress scores showed a substantial reduction in the experimental group compared to the control group, *t*_(116)_ = −5.496, *p* < 0.001, with a mean difference of −24.215 (95% CI [−32.941, −15.489]), demonstrating a significant decrease in stress levels as a result of the intervention. Mental health outcomes also improved significantly, *t*_(116)_ = −6.006, *p* < 0.001, with a mean difference of −25.714(95% CI [−34.194, −17.234]), suggesting that the mindfulness program effectively enhanced the mental health of participants. Furthermore, coping self-efficacy scores increased significantly in the experimental group, *t*_(116)_ = 5.798, *p* < 0.001, with a mean difference of 12.660 (95% CI [8.336, 16.985]), indicating enhanced self-efficacy in managing stress. Lastly, mindfulness levels showed a significant increase, *t*_(116)_ = 6.609, *p* < 0.001, with a mean difference of 7.466 (95% CI [5.2299.703]), underscoring the efficacy of the mindfulness training in fostering a heightened state of mindfulness among the participants.

Overall, the results suggest that the mindfulness intervention was successful in reducing occupational stress and improving mental health, coping self-efficacy, and mindfulness levels among junior high school teachers. These findings provide strong support for implementing mindfulness-based programs to enhance teacher wellbeing and stress management capabilities.

## 5 Discussions and conclusion

This study investigated the effects of a mindfulness-based intervention on coping self-efficacy, mental health, occupational stress, and mindfulness levels among junior high school teachers in China. The findings demonstrated that the intervention group experienced significant improvements across these dimensions, whereas the control group showed no such changes. These results highlight the potential of mindfulness-based programs to alleviate occupational stress and improve teachers' psychological wellbeing.

### 5.1 Mindfulness and teacher wellbeing

Consistent with prior studies (e.g., Janssen et al., 2023; Morales-Rodríguez and Morales-Rodríguez, 2024), the current findings reinforce the effectiveness of mindfulness in enhancing mental health and reducing occupational stress among educators. However, this study contributes a novel dimension by focusing on junior high school teachers in China, a group underrepresented in the literature, thereby addressing a critical cultural and professional gap. Moreover, by tailoring the mindfulness intervention to the specific stressors faced by this population, such as teacher-student relationships and self-growth challenges, the research extends the applicability of mindfulness beyond generic stress-reduction programs.

Unlike traditional interventions that primarily target stress reduction, this study demonstrated that mindfulness training significantly enhances coping self-efficacy. This finding aligns with previous research suggesting that mindfulness can promote emotional regulation and psychological resilience (von Muenchhausen et al., 2021; Tarrasch et al., 2020). However, the integration of compassion-focused practices in the intervention further amplifies its impact, distinguishing this study as one of the few to explore how mindfulness can directly strengthen coping self-efficacy in educational settings.

### 5.2 Occupational stress under mindfulness interventions

The study's division of occupational stress into components such as self-growth, teacher-student relationships, colleague relationships, and work pressure provides a nuanced understanding of the stressors faced by junior high school teachers. While previous research (e.g., Fitchett et al., 2019; Jõgi et al., 2022) has highlighted the complexity of occupational stress, this study uniquely demonstrates how mindfulness interventions can effectively address each stressor. Specifically, the program's focus on self-awareness and emotional regulation significantly reduced stress in both interpersonal and work-related domains.

The successful application of the ADDIE framework to develop and implement the mindfulness program underscores the importance of tailored intervention design. By aligning the intervention with the specific needs of junior high school teachers, the study validates the utility of structured design processes in creating impactful stress management programs (Cotter et al., 2023).

### 5.3 Theoretical and practical implications for guidance

The study's 4-week intervention duration proved was efficient and manageable, attracting greater teacher participation. The combination of online and offline formats facilitated frequent practice and improved mental health outcomes, supporting Regan et al.'s (2020) assertion that high-frequency engagement is crucial for developing mindfulness habits. The reproducibility of the pre-recorded audio and basic mindfulness techniques further enhances the programme's scalability and potential for widespread adoption.

The results suggest that mindfulness training should be integrated into pre-service and in-service teacher education programs to prepare educators with essential stress management tools. Regular mindfulness workshops or certification programs could enhance teachers' coping abilities and resilience, especially in high-stress environments. Group-based mindfulness sessions can also foster a sense of community among teachers, promoting shared experiences and support networks that alleviate occupational stress. Schools could implement structured collaborative psychoeducational programs to enhance not only individual wellbeing but also team dynamics and organizational culture. Given the programme's focus on enhancing self-awareness and emotion regulation, these techniques can also be leveraged to improve teacher-student relationships, reducing conflict and fostering a more harmonious classroom climate. Embedding mindfulness practices in classroom management training can help educators respond to challenges with greater emotional regulation and empathy.

### 5.4 Limitations and future directions

While the study demonstrated the effectiveness of mindfulness interventions, there is potential to enhance mindfulness techniques further and diversify approaches beyond compassionate meditation. Future research should explore various mindfulness methods and their impact on occupational stress to refine and optimize intervention programmes. An in-depth exploration of these techniques will contribute to the continuous improvement of mindfulness curricula and their role in stress management for educators.

The importance of stress reduction efforts in the education sector cannot be overstated. Promoting mindfulness education and practice among junior high school teachers can significantly impact their mental health and the stability of the education system. Future initiatives should focus on disseminating mindfulness knowledge and techniques to foster a positive educational environment, enhance teacher wellbeing, and improve overall educational outcomes.

This study paves the way for several promising research directions in educational psychology, particularly in understanding mechanisms of mindfulness in stress reduction, technology-enhanced mindfulness interventions, and comparative effectiveness of intervention designs. Future research should also explore how integrating mindfulness practices into school-wide psychoeducational models influences institutional wellbeing and organizational culture. Investigating how mindfulness mediates factors like job satisfaction, burnout, and professional identity could provide further insights into its transformative potential in educational settings. Additionally, longitudinal studies examining the cumulative effects of sustained mindfulness practices on both teacher performance and student outcomes could contribute to the development of robust, evidence-based psychoeducational frameworks for stress management.

## Data Availability

The original contributions presented in the study are included in the article/Supplementary material, further inquiries can be directed to the corresponding author.

## References

[B1] Ansley B. Houchins D. Varjas K. Roach A. Patterson D. Hendrick R. (2021). The impact of an online stress intervention on burnout and teacher efficacy. Teach. Teach. Educ. 98:103251. 10.1016/j.tate.2020.103251

[B2] Bandura A. Barbaranelli C. Caprara G. V. Pastorelli C. (2001). Self-efficacy beliefs as shapers of children's aspirations and career trajectories. *Child Dev*. 72, 187–206. 11280478 10.1111/1467-8624.00273

[B3] Benevene P. De Stasio S. Fiorilli C. (2020). The well-being of school teachers in their work environment. Front. Psychol. 11:1239. 10.3389/fpsyg.2020.01239 32903655 PMC7438736

[B4] Bergomi C. Tschacher W. Kupper Z. (2013). The assessment of mindfulness with self-report measures: existing scales and open issues. Mindfulness 4, 191–202. 10.1007/s12671-012-0110-9

[B5] Bishop S. R. Lau M. Shapiro S. Carlson L. Anderson N. D. Carmody J. Devins G. (2004). Mindfulness: a proposed operational definition. Clin. Psychol. Sci. Pract. 11, 230–241. 10.1093/clipsy.bph077

[B6] Candeias A. A. Galindo E. Reschke K. Bidzan M. Stueck M. (2024). The interplay of stress, health, and well-being: unraveling the psychological and physiological processes. Front. Psychol. 15:1471084. 10.3389/fpsyg.2024.1471084 39253034 PMC11381401

[B7] Chennoufi L. Ellouze F. Cherif W. Mersni M. M'rad M. F. (2012). Stress and burnout among Tunisian teachers. L'Encephale 38, 480–487. 10.1016/j.encep.2011.12.012 23200614

[B8] Clunies-Ross P. Little E. Kienhuis M. (2008). Self-reported and actual use of proactive and reactive classroom management strategies and their relationship with teacher stress and student behaviour. *Educ. Psychol*. 28, 693–710.

[B9] Cotter S. Yamamoto J. Stevenson C. (2023). A systematic characterisation of food safety training interventions using the analyse, design, develop, implement, and evaluate (ADDIE) instructional design framework. Food Control 145:109415. 10.1016/j.foodcont.2022.109415

[B10] Dave D. McClure L. Rojas S. De Lavalette O. Lee D. (2020). Impact of mindfulness training on the well-being of educators. J. Altern. Complement. Med. 26, 645–651. 10.1089/acm.2019.0451 32453627

[B11] Denuwara B. Gunawardena N. Dayabandara M. Samaranayake D. (2021). A systematic review and meta-analysis of the effectiveness of individual-level interventions to reduce occupational stress perceptions among teachers. Arch. Environ. Occup. Health 77, 530–544. 10.1080/19338244.2021.1958738 34338619

[B12] Eze A. Anyebe M. Nnamani R. Nwaogaidu J. Mmegwa P. Akubo E.. (2023). Online cognitive-behavioral intervention for stress among English as a second language teachers: implications for school health policy. Front. Psychiatry 14:1140300. 10.3389/fpsyt.2023.1140300 38094591 PMC10716503

[B13] Fitchett P. McCarthy C. Lambert R. Eyal M. Playfair E. Dillard J. (2019). Examining teacher stress-vulnerability in the US secondary school context. Educ. Rev. 73, 170–193. 10.1080/00131911.2019.1619521

[B14] Gluschkoff K. Elovainio M. Kinnunen U. Mullola S. Hintsanen M. Keltikangas- Järvinen L.. (2016). Work stress, poor recovery and burnout in teachers. Occup. Med. 66, 564–570. 10.1093/occmed/kqw086 27412428

[B15] Hoferichter F. Jentsch A. (2024). The effects of an online positive psychology intervention on pre-service teachers' efficacy, ability to cope, and emotional regulation. Br. Educ. Res. J. 50, 2441–2460. 10.1002/berj.4036

[B16] Janssen M. Heerkens Y. Van der Heijden B. Korzilius H. Peters P. Engels J. (2023). Effects of mindfulness-based stress reduction and an organisational health intervention on Dutch teachers' mental health. Health Promot. Int. 38:daac008. 10.1093/heapro/daac008 35134930 PMC10313345

[B17] Jõgi A. Aulén A. Pakarinen E. Lerkkanen M. (2022). Teachers' daily physiological stress and positive affect in relation to their general occupational well-being. Br. J. Educ. Psychol. 93, 368–385. 10.1111/bjep.12561 36336902 PMC10098726

[B18] Kabat-Zinn J. (1994). Where you go, there you are: Mindfulness meditation in everyday life. New York, NY: Hyperion.

[B19] Kabat-Zinn J. (2003). Mindfulness-based interventions in context: Past, present, and future. *Clin. Psychol. Sci. Pract*. 10, 144–156. 10.1093/clipsy.bpg016

[B20] Kim L. E. Jörg V. Klassen R. M. (2019). A meta-analysis of the effects of teacher personality on teacher effectiveness and burnout. Educ. Psychol. Rev. 31, 163–195. 10.1007/s10648-018-9458-2 30930595 PMC6407857

[B21] Mikus K. Teoh K. (2021). Psychological Capital, future-oriented coping, and the well-being of secondary school teachers in Germany. Educ. Psychol. 42, 334–353. 10.1080/01443410.2021.1954601

[B22] Montgomery C. Rupp A. A. (2005). A meta-analysis for exploring the diverse causes and effects of stress in teachers. Can. J. Educ. 28, 458–486. 10.2307/4126479

[B23] Morales-Rodríguez A. M. Morales-Rodríguez F. M. (2024). Effectiveness of a mindfulness-based intervention program to improve communication and stress coping skills in university students. Eur. J. Investig. Health Psychol. Educ. 14, 1927–1939. 10.3390/ejihpe14070128 39056643 PMC11275262

[B24] Nevill R. E. Havercamp S. M. (2019). Effects of mindfulness, coping styles and resilience on job retention and burnout in caregivers supporting aggressive adults with developmental disabilities. J. Intellect. Disabil. Res. 63, 441–453. 10.1111/jir.12594 30687982

[B25] Nwoko J. Emeto T. Malau-Aduli A. Malau-Aduli B. (2023). A systematic review of the factors that influence teachers' occupational wellbeing. Int. J. Environ. Res. Public Health 20:6070. 10.3390/ijerph20126070 37372657 PMC10298565

[B26] Paudel N. Adhikari B. Prakash K. Kyrönlahti S. Nygård C. Neupane S. (2022). Effectiveness of interventions on the stress management of schoolteachers: a systematic review and meta-analysis. Occup. Environ. Med. 79, 477–485. 10.1136/oemed-2021-108019 35256508

[B27] Rashid A. A. A. (2022). The prevalence of psychological stress and its associated factors among junior high school teachers in Samarahan and Asajaya district. Med. Health 17, 180–197. 10.17576/MH.2022.1701.14

[B28] Regan T. Harris B. Van Loon M. Nanavaty N. Schueler J. Engler S.. (2020). Does mindfulness reduce the effects of risk factors for problematic smartphone use? Comparing the frequency of use versus self-reported addiction. Addict. Behav. 108:106435. 10.1016/j.addbeh.2020.106435 32335396

[B29] Rey L. Extremera N. Pena M. (2016). Emotional competence relating to perceived stress and burnout in Spanish teachers: a mediator model. PeerJ 4:e2087. 10.7717/peerj.2087 27280077 PMC4893324

[B30] Samfira E. Paloş R. (2021). Teachers' personality, perfectionism, and self-efficacy as predictors for coping strategies based on personal resources. Front. Psychol. 12:751930. 10.3389/fpsyg.2021.751930 34795619 PMC8593193

[B31] Schwarzer R. Hallum S. (2008). Perceived teacher self-efficacy as a predictor of job stress and burnout: mediation analyses. *Appl. Psychol*. 57, 152–171. 10.1111/j.1464-0597.2008.00359.x

[B32] Segal Z. V. Teasdale J. D. Williams J. M. Gemar M. C. (2002). The mindfulness-based cognitive therapy adherence scale: Inter-rater reliability, adherence to protocol and treatment distinctiveness. *Clin. Psychol. Psychother*. 9, 131–138.

[B33] Shapiro S. L. Carlson L. E. Astin J. A. Freedman B. (2006). Mechanisms of mindfulness. *J. Clin. Psychol*. 62, 373–386. 16385481 10.1002/jclp.20237

[B34] Starr E. J. (2023). Job stress moderates the effects of a mindfulness intervention on early childhood teachers' emotion dysregulation (master's thesis). University of Nebraska-Lincoln, Lincoln, NE, United States.

[B35] Sun J. Wang Y. Wan Q. Huang Z. (2019). Mindfulness and special education teachers' burnout: the serial multiple mediation effects of self-acceptance and perceived stress. Soc. Behav. Pers. 47:e8656. 10.2224/sbp.8656

[B36] Tarrasch R. Berger R. Grossman D. (2020). Mindfulness and compassion as key factors in improving teacher's well-being. Mindfulness 11, 1049–1061. 10.1007/s12671-020-01304-x

[B37] Tesfaye A. Abate K. Kabito G. Azale T. (2023). Perceived occupational stress and associated factors among primary school teachers in the second wave of COVID-19 in Ethiopia: a multicenter cross-sectional survey. Front. Public Health 11, 1–8. 10.3389/fpubh.2023.1156652 37404268 PMC10315464

[B38] Tsang K. Shum K. Chan W. Li S. Kwan H. Su M.. (2021). Effectiveness and mechanisms of mindfulness training for school teachers in difficult times: a randomized controlled trial. Mindfulness 12, 2820–2831. 10.1007/s12671-021-01750-1 34545293 PMC8443903

[B39] von Muenchhausen S. Braeunig M. Pfeifer R. Göritz A. Bauer J. Lahmann C.. (2021). Teacher self-efficacy and mental health—their intricate relation to professional resources and attitudes in an established manual-based psychological group program. Front. Psychiatry 12:510183. 10.3389/fpsyt.2021.510183 34122155 PMC8193039

[B40] Wang Y. Ramos A. Wu H. Liu L. Yang X. Wang J. Wang L. (2015). Relationship between occupational stress and burnout among Chinese teachers: A cross-sectional survey in Liaoning, China. *Int. Arch. Occup. Environ. Health* 88, 589–597. 25256806 10.1007/s00420-014-0987-9

[B41] Wettstein A. Gabriel J. Kühne F. Grosse Holtforth M. La Marca R. (2023). Predictors of psychological strain and allostatic load in teachers: examining the long-term effects of biopsychosocial risk and protective factors using a LASSO regression approach. Int. J. Environ. Res. Public Health 20:5760. 10.3390/ijerph20105760 37239489 PMC10218379

[B42] Won S. Chang E. (2019). The relationship between school violence-related stress and quality of life in school teachers through coping self-efficacy and job satisfaction. School Ment. Health 12, 136–144. 10.1007/s12310-019-09336-y

[B43] Zhu C. Liu J. Shen J. (2002). Research on the sources of occupational stress among primary and junior high school teachers (in Chinese). Mod. Prim. Second. Educ. 3, 50–55.

