# Supplementary material for: Alleviating occupational stress in Chinese junior high school teachers: the role of mindfulness-based interventions
Source: Front Psychol. 2025 Jan 29;16:1479507. doi: 10.3389/fpsyg.2025.1479507 (PMC11813917; doi:10.3389/fpsyg.2025.1479507)
Supplement: Supplementary file 1 [file Table_1.docx]

# Appendix 1

**Mindfulness Actions in the Course**

| **Approaches** | **Descriptions** | **Body position** |
| --- | --- | --- |
| Mindfulness of Breathing | Focusing on one's breath allows an individual to perceive each breath in and out. By observing the breath, people can develop concentration, deepen their breathing, lower their heart rate, and find peace in times of stress and anxiety. | Sitting down., Lying down |
| Mindfulness of Walking | Mindfulness of Walking is a mindfulness exercise that takes the feeling of walking as the object of observation. During practice, one should pay attention to the feeling of contact between the soles of the feet and the ground or the lifting, moving, and lowering of the feet during walking. One can also focus on the feeling of the soles of the feet, calves, and thighs. | Walking |
| Observing emotions | By observing emotions, individuals learn to recognize, accept, and understand them rather than being controlled by them. This helps increase emotional intelligence, reduce anxiety and emotional stress, and better deal with emotions. | Sitting down |
| Sitting meditation | By focusing on breath and bodily sensations, individuals cultivate awareness of the present moment, learning to face inner thoughts and feelings with a nonjudgmental, nonreactive attitude. This helps enhance self-awareness and reduce psychological tension, enabling individuals to face inner fluctuations more calmly. | Sitting down |
| Loving-kindness meditation | Loving-kindness meditation is a Mindfulness practice that cultivates compassion. The practice should involve a series of blessings for different objects in a certain order. The blessings include words of peace, health, freedom from suffering, joy, and love. These blessings can be given to the practitioners themselves, their benefactors, loved ones, other ordinary people, or even people they don't like. | Lying down. Sitting down. |

**APPENDIX 2**

**The Contents of the Mindfulness Course**

| **Week** | **Week Objectives** | **Activity** |
| --- | --- | --- |
| Week 1:  Self-acceptance | Intention： to cultivate self-acceptance, reduce self-demands and self-criticism;  Attention： to focus on inner experiences and enhance self-awareness;  Attitude： to approach emotions openly, promote healthy self-acceptance; | 1. "Greeting with Mindfulness”;  2. "Dancing With Feelings ";  3. Loving-kindness meditation: self-acceptance;  4. Mindfulness with practice and Q&A (Mindfulness of breathing)  Daily activity for the week: Mindfulness of Breathing |
| Week 2:  Caring students | Intention： to enhance teacher-student interaction, to improve teacher-student relationships;  Attention： to focus on teacher-student interaction and understand student needs;  Attitude： to treat students kindly and build positive relationships; | 1. "T-S Exercise";  2. "Me in the eyes of students";  3. Loving-kindness meditation: Caring students;  4. Mindfulness with practice and Q&A（Sitting meditation）  Daily practice of Sitting meditate  Equipment： Paper and pen |
| Week 3  Cherish Colleagues | Intention： to foster collaboration and enhance colleague relationships;  Attention： to focus on colleague interactions and strengthen communication;  Attitude： To respect colleagues and promote harmonious relationships | 1. "Eye-to-eye observation";  2. " My Support Circle ".  3. Loving-kindness meditation: Cherishing Colleagues;  4. Mindfulness with practice and Q&A（Mindfulness of emotions）  Daily practice of observing emotions  Equipment: Paper and pen |
| Week 4  loving work | Intention： to address work pressure and find meaning in work;  Attention： to focus on tasks, alleviate pressure and anxiety;  Attitude： To cope positively with work pressure and lighten the emotional burden. | 1. "Self-encouragement";  2. "Growth nodes";  3. Loving-kindness meditation: love of work.  4. Mindfulness with practice and Q&A（Mindfulness of walking）  Daily Practice of Mindfulness of walking  Equipment： Paper and pen |
